# Correlative x-ray microscopy and transmission electron microscopy for ultrastructural analysis of zebrafish tissue

**DOI:** 10.1186/s42649-025-00120-8

**Published:** 2025-12-27

**Authors:** Sun-Yeong Gwon, MinKi Choi, Ji Young Mun

**Affiliations:** 1 Neural Circuit Research Group, Korea Brain Research Institute, Daegu, 41068 Republic of Korea; 2 Research Microscopy Solutions, Carl Zeiss Co, Ltd, Seoul, 05836 Republic of Korea

**Keywords:** Volume electron microscopy, X-ray microscopy, Transmission electron microscopy, Correlative microscopy, Zebrafish, Neuromast, Ultrastructure

## Abstract

We established a correlative workflow combining X-ray microscopy (XRM) and transmission electron microscopy (TEM) to investigate the ultrastructure of zebrafish neuromasts. Zebrafish tissues were processed using conventional TEM embedding protocols and first imaged with XRM to obtain three-dimensional information and to localize the neuromast within resin blocks. The XRM previews enabled accurate trimming and rapid identification of target regions, reducing the need for repeated semi-thin sectioning and toluidine blue staining. Subsequent TEM analysis revealed ultrastructural details including cell-to-cell contacts and organelle morphology. Our study demonstrates that integration of XRM with TEM increases efficiency, minimizes tissue loss, and improves orientation accuracy for ultrastructural analysis. This correlative approach provides a valuable workflow for volume electron microscopy, applicable to the observation of the neuromast and various other tissues.

## Introduction

X-ray microscopy (XRM) generates high-resolution, non-destructive images of biological samples, enabling three-dimensional (3D) visualization of thick tissues. Advances in XRM have extended its application to materials science, chemistry, and biology, providing structural information at nanometer scales. In biological research, XRM has been particularly valuable for visualizing tissue organization and guiding subsequent high-resolution imaging (Lider 2017; Bushong et al. 2020). After Röntgen’s discovery of X-rays, the X-ray projection microscope was developed to enable biological research at nanometer resolution (Duncumb, 2004). In particular, XRM allows non-destructive imaging of biological samples, providing tissue organization through 3D visualization of thick tissues (Lider 2017; Ahn, et al., 2025). Advances in XRM, particularly when combined with spectroscopic microscopy, have expanded its applications in materials science, chemistry (Gilbert 1972; Dubochet 1988), and biology (Shearer et al., 2016; Duncan et al. 2022). This integration enables comprehensive chemical analysis (Thieme et al. 2010; Nazaretski et al. 2019, 2021), providing insights into elemental composition, crystalline phases, and structural details at the nanometer scale (Feser, 2015).

Transmission X-ray microscopy provides high-resolution images through full-field projection with a single exposure, enabling integration with computed tomography to obtain 3D sample information (Yuan et al. 2021). A water-window X-ray microscope combined with wide-field transmission microscopy further allows acquisition of both two-dimensional and 3D structures (Ohsuka et al. 2016). In biology, X-ray micro-tomography techniques such as scanning transmission X-ray microscopy and X-ray fluorescence microspectroscopy reveal elemental distributions within samples, which is particularly useful for studying intracellular mechanisms and nanoparticle interactions (Leung et al. 2010; Rio-Echevarria et al. 2019). In addition, integrating XRM with serial block-face scanning electron microscopy enhances region-of-interest tracking and improves targeting efficiency for electron microscopy (Bushong et al. 2014, 2020).

While SEM has seen significant improvements in resolution, many researchers still prefer TEM due to its better resolution and other applications. However, one of the persistent challenges in TEM workflows is achieving precise trimming and sectioning of the sample, which remains a critical bottleneck for many users. Nevertheless, these two techniques are complementary: XRM is advantageous for imaging thick samples, while TEM offers high spatial resolution for ultrastructure of cells.

The objective of this study was to examine the intracellular ultrastructure of the zebrafish neuromast. In this study, we applied both methods to analyze the neuromast of zebrafish specimens embedded in resin blocks. While conventional TEM protocols are labor-intensive, XRM provides substantial time savings and enables 3D reconstruction. Thus, integrating XRM with TEM improves efficiency and yields more comprehensive structural information.

## Materials and methods

### TEM sampling

The zebrafish tissues provided from KRIBB fixed with 2% Paraformaldehyde and 2.5% glutaraldehyde and cut. The tissues were processed according to the NCMIR methods for 3D EM (Deerinck et al. 2010, 2022; Jung and Mun 2019). The methods were described as follows: Tissues were washed for 5 min three times in cold cacodylate buffer. Then, 3% potassium ferrocyanide in 0.3 M cacodylate buffer was combined with an equal volume of 4% aqueous osmium tetroxide (final concentration was 1.5% potassium ferrocyanide and 2% aqueous osmium tetroxide in 0.15 M cacodylate buffer). The tissues were incubated in this solution on ice for 1 h. During incubation time, 0.1 g thiocarbohydrazide (TCH) was dissolved 10 mL ddH2O and placed in a 60℃ oven for 1 h and gently swirling every 10 min. Before use, TCH solution was filtered through a 0.22 um Millipore syringe filter. After osmium incubation, the tissues were washed with ddH2O at room temperature for 5 min three times. The tissues were placed in the filtered TCH solution for 20 min at room temperature. Then the tissues were rinsed in ddH2O for 5 min three times, at room temperature. After this step, they were placed in 2% osmium tetroxide in ddH2O for 30 min, at room temperature. Following this step, the tissues were washed 5 min three times at room temperature in ddH2O then in 1% uranyl acetate at 4℃ overnight. The tissues were washed ddH2O as same ways ahead and placed in lead aspartate solution at 60℃ oven for 30 min. Then, tissues were washed by ddH2O and dehydrated using ice-cold solutions of 20%, 50%, 70%, 90%, 100%, 100% ethanol (anhydrous), 10 min each, then placed in anhydrous ice-cold 100%, 100% acetone and left at room temperature for 10 min each. During that time, hard resin (EMbed 812 Resin, EMS, #14,120) was formulated by weight and mixed with acetone by concentration. Then the tissues were placed into 25% resin: Acetone for 2 h, then into 50% resin: Acetone for 2 h and 75% resin: Acetone for 2 h. Tissues were placed in 100% resin overnight then into fresh 100% resin for 2 h. The tissue pieces were embedded in a rein in a silicon mold and placed in a 60℃ oven for 48 h.

### XRM analysis

Resin-embedded zebrafish specimens were scanned with a 20 × objective lens, producing a voxel size of 0.6 µm under Xradia 620 Versa system (Carl Zeiss, Germany, installed at Zeiss innovation center, Dongtan, Korea). The X-ray source was operated at 80 kV and 10 W, and a total of 4,501 projection images were acquired over a rotation angle of 180° with fan mode. Each projection was recorded with an exposure time of 1 s, and an LE4 filter was applied during imaging. The total scan duration was approximately 4.5 h. Reconstruction of the acquired datasets was performed using DeepRecon Pro, a machine learning–based algorithm for noise reduction, to enhance image quality and facilitate 3D visualization of the specimens. To visualize and analyze the images, Dragonfly software program was used.

### TEM analysis

The resin block was prepared following the conventional TEM methods (Porter and Blum 1953; Winey et al. 2014; Jung and Mun 2019). After TEM sampling, the resin block was trimmed by razor blades for removing the excess resin without tissue. Then the 500 nm semi-thin section was performed using an ultramicrotome. The slices on the glass slide were stained by toluidine blue (TB) to distinguish whether there was a target or not. After finding the target, the resin block was sectioned by ultramicrotome 70–100 nm and the slices put on the formvar coated-one hole grid. Then the grid was observed under TEM at 120 kV. The TEM images were analyzed using DigitalMicrograph® software made by Gatan, Inc. and ImageJ Fiji from NIH.

## Results and discussion

**Fig. 1:**
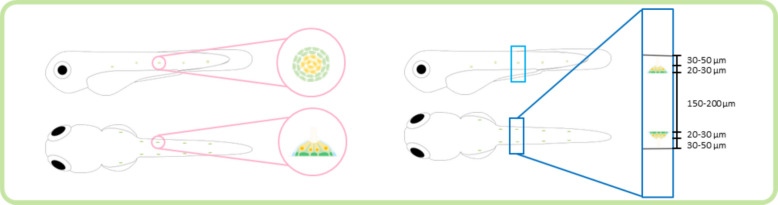
Neuromast of the zebrafish. The schematic shows the position and orientation of the neuromast when viewed from the lateral side, and the lower schematic shows the neuromast from the dorsal view. The pink circle marks the cross-sectional area of the neuromast. The sky-blue and blue boxes indicate the trimming regions used for TEM sampling, with the blue box extended dorsally to represent the depth of the neuromast within the tissue

The zebrafish neuromast is located laterally between the dorsal and ventral sides of the zebrafish. It measures approximately 20–30 µm in diameter with a depth of 30–50 µm from the skin surface (Fig. 1) and is composed of hair cells (yellow cells), supporting cells (green cells), and mantle cells (blue cells). These sensory organs detect water flow through ciliary motion, providing essential mechanosensory functions (López-Schier et al. 2004; Dow et al. 2018).

### Approaches for target localization in biological samples

**Fig. 2:**
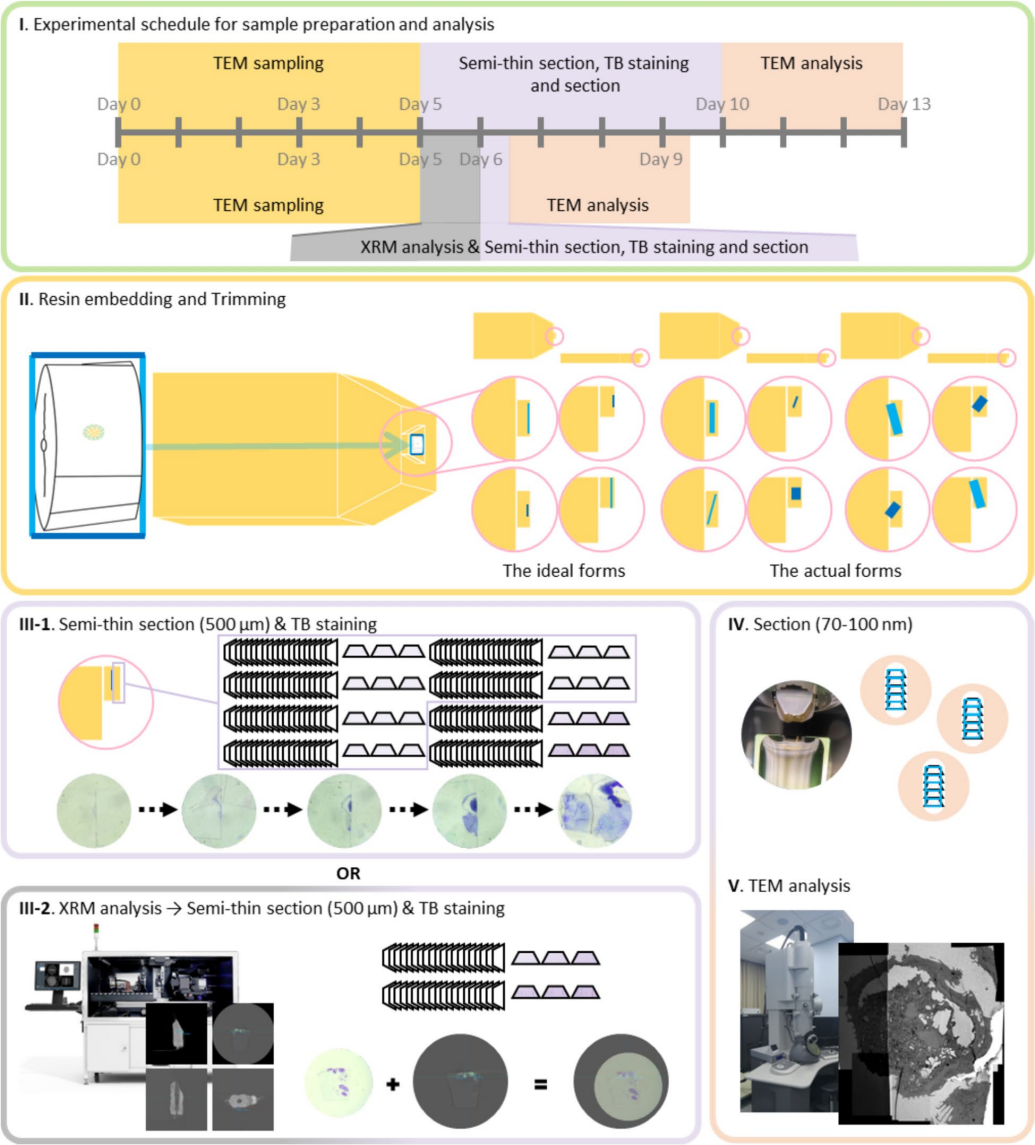
Technical procedure of sample preparation and analysis. I. Workflow of sample preparation and analysis for a single specimen. II. Zebrafish sample prepared for TEM; the schematic shows the ideal and actual shapes after resin embedding. The sky-blue and blue lines indicate the tilt of the tissue relative to the resin block surface. III-1. Conventional method: serial semi-thin sectioning with TB staining. III-2. XRM-assisted method: 3D imaging to determine the depth and orientation of the target, followed by semi-thin sectioning and TB staining. IV–V. Ultrathin sectioning and TEM observation

Specimens were fixed with glutaraldehyde, paraformaldehyde, ferrocyanide-reduced osmium tetroxide, and thiocarbohydrazide–osmium ligation staining. Following dehydration and embedding in resin, the neuromast was difficult to identify under light microscopy due to osmium staining. Conventional TEM technique required semi-thin sectioning and TB staining for search the location to study, which is labor-intensive and time-consuming. As illustrated in Fig. 2I, the EM sampling, semi-thin sectioning, TB staining, sectioning, and TEM analysis were conducted in the same manner. The duration of these processes depended on how deeply the target tissue was located within the resin block and on its orientation relative to the block surface. The ideal embedded tissue was oriented parallel to the anterior surface of the resin block, with a minimal distance from the front surface to the target (Fig. 2II). However, most embedded tissues were tilted. The conventional method (Fig. 2III-1), which required slice-by-slice confirmation, was time-consuming and demanded considerable effort to accurately locate the neuromast. As illustrated in Fig. 2III-1, TB staining was performed at intervals of every tenth to twentieth slice. Because slight changes in sectioning angle can markedly affect TEM images, it is essential to precisely control the angle during resin block sectioning. By applying XRM prior to trimming, we were able to generate 3D images that revealed the depth, orientation, and morphology of the neuromast (Fig. 2III-2). This information enabled accurate trimming and successful semi-thin sectioning with TB staining.

Following TB staining, ultrathin Sects. (70–100 nm) were cut using an ultramicrotome (Fig. 2IV). Each section was placed on a formvar-coated one-hole grid and observed under TEM (Fig. 2V).

### Correlation of XRM and TEM imaging

**Fig. 3:**
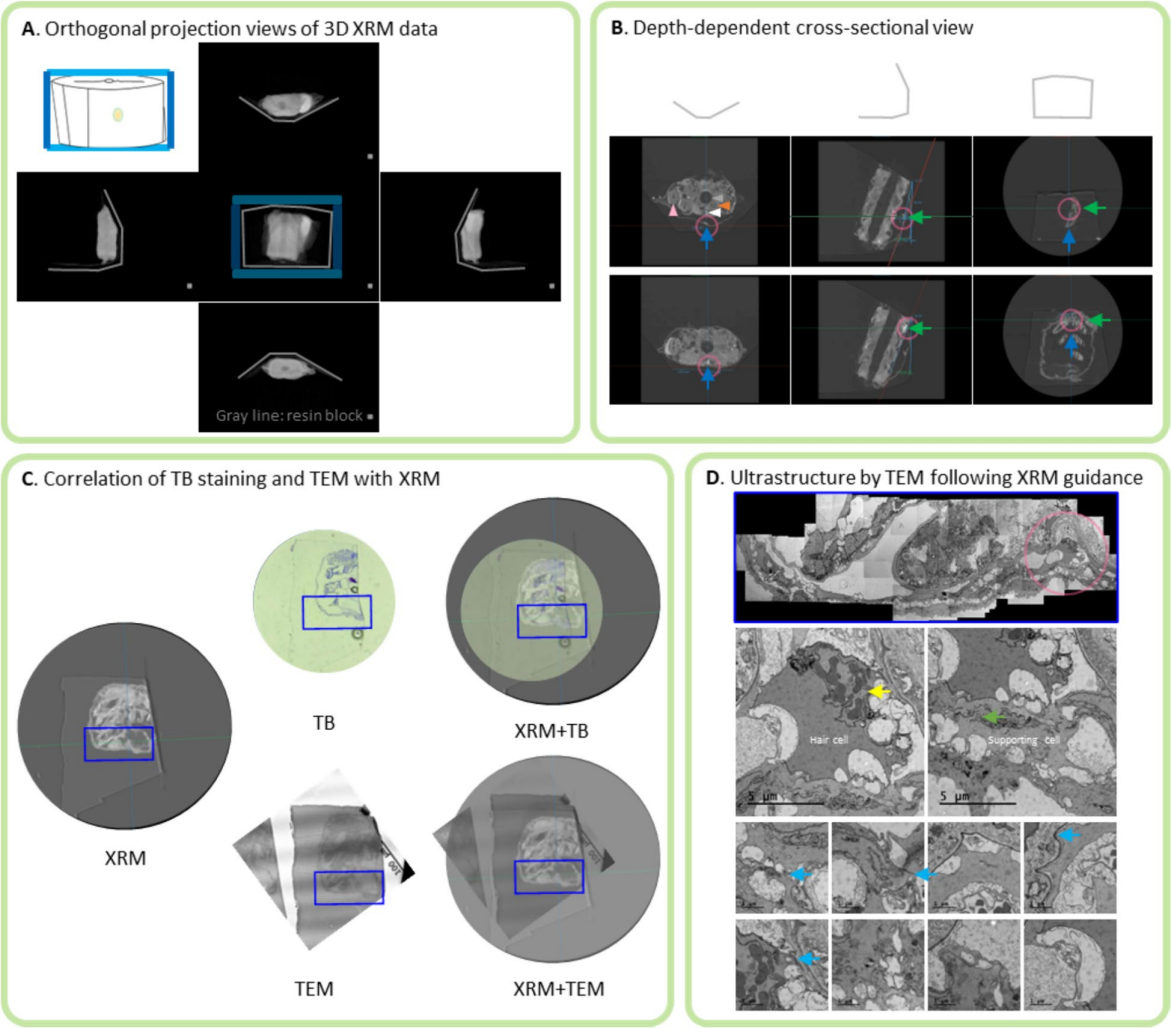
Neuromast images obtained by XRM and TEM. **A** 3D XRM projection image of the zebrafish neuromast in planar view. The blue box corresponds to the same orientation. The view in each orientation shows the resin block. The grey lines represent the boundaries of the resin block as seen in the X-ray. **B** XRM cross-sectional images at different depths and angles. The left, middle, and right panels show views from the top, side, and front, respectively. The pink circle indicates the neuromast. Specific tissue structures are indicated by arrowheads: muscle tissue (orange arrowhead), neurons (white arrowhead), notochord (grey arrowhead) and the yolk sac (pink arrowhead). **C** and **D** Localization of the neuromast confirmed by semi-thin sectioning and TB staining, followed by TEM. The circled images show XRM and TB-stained sections. The large stitched TEM image (blue box, 1700 × magnification) and higher magnification TEM images reveal ultrastructural details, with the pink circle marking the neuromast. Subcellular details are further highlighted by arrows: the nucleus (yellow arrow), mitochondria (green arrow), and cell-to-cell interaction (blue arrow)

The 3D XRM reconstructions provided clear visualization of tissue structures within the resin block, and the rotation tool in Dragonfly software confirmed the integrity of the 3D structural representation (Fig. 3A). The rotation tool showed images of the anterior tissue in the resin block from the front, right, left, top, and bottom views. These images illustrated the tissue’s position within the resin block and its distance from the surface. The 3D X-ray images revealed muscle, neurons, notochord and yolk sac, which were readily distinguished. The neuromast appeared brighter than surrounding tissues and showed distinctive morphology, allowing clear identification. Neuromasts are arranged along the lateral line in zebrafish and are innervated by lateral line nerves, which are known to be myelinated. Given that myelin exhibits higher X-ray intensity, it is plausible that the bright area observed beneath the neuromast corresponds to the myelinated lateral line nerve. In addition, cross-sectional views along the X, Y, and Z axes facilitated accurate prediction of the neuromast position (Fig. 3B). These predictions closely matched the subsequent TB-stained sections, confirming the reliability of XRM for pre-localization (Fig. 3C). By adjusting the block angle in the software, we could preview the sections expected after semi-thin sectioning, allowing optimal orientation for targeting. Distance and angle data from XRM enabled precise semi-sectioning at the target location, greatly improving efficiency compared with conventional methods. Even when the preview did not perfectly align, it still allowed estimation of the target’s position and tilt. After TB staining, ultrathin sections were prepared, mounted on formvar-coated one-hole grids, and observed under TEM. The XRM previews correlated with the TEM images (Fig. 3C), and TEM images provided high-resolution ultrastructure of the neuromast, including hair cells and supporting cells, cell-to-cell contacts, and intracellular organelles (Fig. 3D).

### Advantages of combined XRM–TEM workflow

The integration of XRM with conventional TEM preparation workflows offers several advantages. First, it reduces the time and effort required for target identification within resin-embedded tissues. Second, it provides 3D orientation data, which helps minimize sectioning errors and prevents loss of the target tissue. Finally, it ensures that TEM can be focused on the precise region of interest, thereby enhancing efficiency and data quality. This correlative approach demonstrates the value of XRM as a complementary tool to TEM for ultrastructural studies of small, deeply embedded targets such as the zebrafish neuromast.

## Conclusion

XRM provides 3D volume data of resin-embedded tissues, allowing rapid and accurate localization of target structures. Integration with TEM enhances efficiency, reduces sample loss, and improves orientation accuracy for ultrastructural studies. This correlative workflow represents a powerful tool for volume microscopy in biology.

## Data Availability

The datasets used and/or analyzed during the current study are available from the corresponding author on reasonable request.

## Abbreviations

XRM: X-ray microscopy
3D: Three-dimensional
TEM: Transmission electron microscopy
TCH: Thiocarbohydrazide
TB: Toluidine blue
